# Personalized Approach for Acne Management With Dermocosmetics According to Initial Skin Sensitivity

**DOI:** 10.1111/jocd.70831

**Published:** 2026-04-08

**Authors:** Ju Hee Han, Hyun Jeong Ju, Tae In Kim, Young Hoon Yoon, Young Jun Woo, Heesang Kye, Jong Yeob Kim, Su Jin Jeong, Eunsun Baek, Jung Min Bae

**Affiliations:** ^1^ Department of Dermatology, College of Medicine The Catholic University of Korea Seoul Korea; ^2^ Dermatology Doctors Dermatology Clinic Seoul Korea; ^3^ Dermatology The Yoon Dermatologic Clinic Seoul Korea; ^4^ Dermatology The Mimodern Dermatologic Clinic Seoul Korea; ^5^ Dermatology KYE‐Skin Clinic Seoul Korea; ^6^ Dermatology Timeless Dermatologic Clinic Seoul Korea; ^7^ Statistics Support Part, Medical Science Research Institute Kyung Hee University Seoul Korea; ^8^ Medical Affairs, La Roche Posay, Loreal Dermatological Beauty L'Oréal Korea Ltd. Seoul Korea

## Abstract

**Background:**

Current acne management relies largely on topical and systemic pharmacotherapy, but these treatments frequently cause cutaneous adverse reactions. Such side effects are of particular concern in patients with initial skin sensitivity, highlighting the need for personalized approaches.

**Methods:**

This 12‐week prospective observational study included 308 Korean acne patients to compare the clinical efficacy of a specific dermo‐cosmetic (DC) formulation used as monotherapy versus combination therapy in acne patients, and to evaluate a sensitivity‐based approach to improve quality of life. Patients were classified into DC monotherapy (*n* = 151) or combination therapy (*n* = 157), and to sensitive (*n* = 144) or non‐sensitive (*n* = 164) subgroups. Primary outcomes were Global Evaluation of Acne (GEA), total lesion count, and sebum secretion levels. Secondary outcomes included skin sensitization score, tolerance assessment, quality of life, and adverse events.

**Results:**

The DC monotherapy group showed significantly greater improvement in GEA scores, fewer sensitivity symptoms, improved quality of life, and greater tolerability than the combination therapy group (all *p* < 0.0001). In the sensitive patient subgroup, DC monotherapy was also significantly superior to combination therapy in terms of GEA, tolerability, sensitivity symptoms, and quality of life (all *p* < 0.0001). In patients without initial sensitivity, improvement in GEA (*p* = 0.0079), total lesion count (*p* < 0.0001), and sebum secretion (*p* < 0.0001) was significantly greater in the combination therapy than in the DC monotherapy.

**Conclusion:**

These findings suggest that baseline skin sensitivity may represent an important determinant of treatment selection and support a sensitivity‐guided personalized treatment approach in acne management.

## 1 Introduction

Acne Vulgaris is a chronic inflammatory dermatological condition resulting from a complex interplay of hyperactive sebaceous glands, abnormal follicular keratinization, proliferation of *Cutibacterium acnes*, and inflammatory responses [1]. It exhibits a high prevalence, affecting approximately 80% of the global population at some point in their lives, and beyond physical discomfort, it leads to psychosocial issues such as depression, anxiety, and a reduced quality of life [1, 2, 3, 4, 5].

Current acne treatment guidelines primarily focus on therapeutic selection, based on lesion severity and type, including topical retinoids, benzoyl peroxide, antibiotics, and other pharmacological agents [6]. However, these standard therapies commonly induce various local tolerability‐related adverse events such as skin dryness, redness, itching, and burning sensations [7]. These adverse events compromise patient adherence and ultimately negatively impact long‐term treatment success rates [8]. Clinical concerns regarding irritant reactions during acne treatment have persistently been raised, particularly in patients with sensitive skin [9]. However, systematic and in‐depth clinical research on how an individual's “initial skin sensitivity level” affects the outcomes and tolerability of specific treatment regimens remains scarce. Furthermore, clear academic evidence is lacking regarding the extent to which sensitivity‐related problems are prominent in patient groups with a history of irritation or adverse reactions from past procedures or product use. Therefore, the importance of evaluating initial skin sensitivity characteristics before initiating treatment and establishing individualized strategies based on this assessment has remained an unmet medical need.

In recent years, the dermo‐cosmetics (DCs) market has grown rapidly, and these products, containing functional active ingredients, have begun to occupy a significant position in acne treatment, extending beyond their auxiliary roles in improving skin barrier function, alleviating inflammation, and managing acne lesions [10, 11, 12]. However, widespread accessibility has led to an increase in cases where general consumers misuse or overuse DC products without professional guidance. This has frequently resulted in skin irritation and exacerbation of existing acne, manifesting as acne cosmetica (acne induced by cosmetics and procedures) [13]. Against this backdrop, establishing proper usage guidelines for DC products, especially for safe and effective application in sensitive skin patients, has become a critical task for the medical community. For patients with a history of adverse events from existing treatments or those with sensitive skin, the necessity of evaluating skin sensitivity before starting treatment is emphasized [12]. Moreover, differentiated management strategies based on sensitivity levels, along with education on selecting reliable dermo‐cosmetic products and proper usage guidelines, are urgently needed.

Addressing these unmet medical needs, this study selected a specific DC formulation as the research subject. This product contains active ingredients like salicylic acid, niacinamide, and phyllobioamine, recommended by the domestic acne treatment guidelines (DC algorithm) [12], and was chosen to verify its safety and efficacy.

Acknowledging the possibility of initial characteristic imbalances among acne patient groups, this study set the patient's “initial skin sensitivity” as the core analytical criterion from the outset. It then compared the effects of DC monotherapy and combination therapy (DC‐integrated conventional therapy with established pharmacological agents). This approach aims to explore how treatment strategies (DC monotherapy vs. combination therapy) based on sensitivity, considering heterogeneous patient characteristics, can offer differential benefits in acne improvement, thereby seeking individualized acne management solutions that overcome the limitations of existing treatments. Through this, we aim to complement existing acne treatment guidelines and, particularly for sensitive acne patients, propose new standard treatment options, thereby laying a crucial academic and medical foundation for ushering in a patient‐centric, personalized acne management era.

## 2 Materials and Methods

### 2.1 Study Design and Participant Recruitment

This research was designed as a multi‐center, prospective, observational study conducted across 10 institutions in South Korea from December 2024 to September 2025. This multi‐center approach, including tertiary, secondary, and primary dermatology institutions, was adopted to comprehensively assess the real‐world efficacy and tolerability of a specific DC regimen, both as a monotherapy and combination therapy, particularly considering different skin sensitivity profiles in patients with acne. The study ultimately enrolled a total of 308 adolescent and adult patients aged 12 years or older, diagnosed with mild to moderate facial acne lesions, who met the predefined inclusion criteria. Patients were excluded from participation if they had severe systemic diseases, were pregnant or lactating, or were planning to initiate or alter other acne‐affecting therapeutic interventions during the study period. The study protocol was reviewed and approved by the Institutional Review Boards of all participating institutions and was conducted in accordance with the principles of the Declaration of Helsinki. Written informed consent was obtained from all participants prior to enrollment.

### 2.2 Management Regimen and Group Stratification

To reflect diverse real‐world clinical scenarios, patients were classified according to two criteria at baseline. The first was treatment regimen—use of a specific dermocosmetic regimen (La Roche‐Posay Effaclar Duo+M routine) as dermocosmetic DC monotherapy versus its integration as an adjunct to conventional acne therapy (combination therapy). The second was initial skin sensitivity status (sensitive vs. non‐sensitive), to assess whether sensitivity‐based treatment strategies provide differential benefits in acne improvement. The DC monotherapy group (*n* = 151) exclusively received a standardized DC skincare regimen, having either discontinued or not used conventional pharmacological agents. The combination therapy (DC‐Integrated conventional therapy group, *n* = 157) received the standard DC skincare regimen alongside conventional acne pharmacotherapy and/or physical procedures. This latter group was further subdivided into the drug combination group (for patients using topical/systemic medications like BPO, retinoids, antibiotics, etc.) and the physical modality combination group (for patients receiving only physical procedures such as laser, peeling, or extractions without medications). A critical aspect of this study was an additional stratification based on the presence or absence of “initial skin sensitivity” at baseline. The sensitive patient subgroup (*n* = 144) comprised individuals with a confirmed history of skin irritation or adverse events. This subgroup included 77 patients on DC monotherapy and 67 patients on combination therapy (29 in the drug combination group and 38 in the physical modality combination group). Conversely, the non‐sensitive patient subgroup (*n* = 164) consisted of patients without confirmed initial skin sensitivity, including 74 patients on DC monotherapy and 90 patients on combination therapy (51 in the drug combination group and 39 in the physical modality combination group).

The presently investigated DC regimen consisted of the La Roche‐Posay Effaclar Duo+M routine (Purifying Foaming Gel and essence) used for 12 weeks. This routine involved the twice‐daily use of the Purifying Foaming Gel for facial cleansing, followed by the application of the Effaclar Duo+M essence. The essence was to be applied once or twice daily, specifically to affected areas of the skin, based on individual tolerability and clinical assessment. Patients were permitted to use additional non‐comedogenic moisturizers, provided they did not contain active ingredients or properties overlapping with the studied regimen, to maintain skin hydration and comfort. This specific DC (DC‐Eff, EFFACLAR Duo+M, La Roche‐Posay Laboratoire Dermatologique, France) contains a carefully selected blend of active ingredients. These include salicylic acid, a keratolytic agent; lipohydroxy acid, niacinamide, 2‐oleamido‐1,3‐octadecanediol, piroctone olamine, zinc gluconate, Phylobioma ( *Punica granatum* pericarp extract), the pre‐ and probiotic Aqua posae filiformis, and La Roche‐Posay thermal water [12].

### 2.3 Evaluation Endpoints

The study spanned a 12‐week period (3 months), with patient evaluations conducted at baseline and at the 3‐month mark. At the initial visit, each participant underwent a comprehensive dermatological examination. Baseline assessments included the Global Evaluation of Acne (GEA), the overall lesion count (non‐inflammatory lesions/comedones and inflammatory lesions/papules, pustules), sebum secretion level (scaled 0–10), and initial skin sensitivity. Patients were further stratified into sensitive and non‐sensitive subgroups based on their history of skin irritation or adverse events for critical baseline assessment.

This study was designed to compare and evaluate the clinical efficacy of each treatment regimen (DC monotherapy and combination therapy) after 3 months, separately for the sensitive skin and non‐sensitive skin groups. The primary outcome measures included: GEA, categorizing overall acne improvement into “significant improvement (≥ 75%)”, “moderate improvement (50%–74%),” “slight improvement (25%–49%),” and “no effect (< 25%)”; change in total acne lesion count, measured the change in the sum of non‐inflammatory lesions (comedones) and inflammatory lesions (papules, pustules) from baseline to 3 months; and sebum secretion level, which was evaluated on a scale from 0 (none) to 10 (very high).

Secondary endpoints were also comprehensively analyzed to assess treatment efficacy and patient experience. Skin sensitivity changes (investigator‐ and patient‐assessed) were evaluated by measuring the change in skin sensitivity scores from baseline to 3 months, scoring individual symptoms such as erythema, scaling, dryness, and stinging on a 0–3 scale. This analysis aimed to characterize the pattern of sensitivity changes with each treatment regimen and to determine whether increased sensitivity, despite successful acne improvement, was associated with a decline in quality of life. Tolerability was assessed by rating the overall tolerability of the DC routine on a 1 (low) to 4 (excellent) scale, in order to clarify how irritation related to the treatment regimen (DC monotherapy vs. combination therapy) or to skin sensitivity status affected product adherence. Quality of life was evaluated using the Cardiff Acne Disability Index (CADI), with changes in CADI scores from baseline to 3 months serving to determine the impact of treatment modality and changes in skin sensitivity, as well as to compare quality‐of‐life outcomes between initially sensitive and non‐sensitive patients according to treatment strategy. In addition, irritation‐related adverse events (adverse skin reactions such as erythema, dryness, pruritus, and stinging) were recorded in terms of type, incidence, and severity over the 3‐month period. This analysis aimed to verify the actual incidence of adverse events in the initial sensitivity versus non‐sensitivity groups, to determine whether adverse events occurred more frequently in patients with a prior history of skin reactions, and to explore the relationship between adverse event occurrence, the degree of acne improvement, and changes in skin sensitivity.

### 2.4 Statistical Analysis

All statistical analyses were performed using SAS 9.4 software (SAS Institute, Cary, NC, USA). Descriptive statistics were used to characterize the study variables. Quantitative variables such as skin sensitivity score, mean, standard deviation, median, and quartile range were calculated. Qualitative variables such as treatment group assignment (monotherapy vs. combination) were summarized using frequency and percentage. Primary endpoints comparing the proportion of patients experiencing changes in skin sensitivity were assessed, and changes in skin sensitivity scores between baseline and Week 12 were assessed using the Wilcoxon signed rank test, a nonparametric test suitable for paired data that may not be normally distributed. Furthermore, for sub‐analysis, a general linear model and a mixed model for repeated measurement analysis were performed to see the effect of the treatment group according to the presence or absence of sensitivity or the combination of drugs and physical modality.

## 3 Results

### 3.1 Study Population and Baseline Characteristics

A total of 308 patients participated in the study. The mean age of patients was 29.5 ± 9 years (range 10–51 years), consisting of 32.8% males (101 patients) and 67.2% females (207 patients). The DC monotherapy group comprised 151 patients (49.0%), and the combination therapy group comprised 157 patients (51.0%) (Table 1A).

**TABLE 1:** Baseline characteristics of study participants.

(A)	Total (*n* = 308)	Monotherapy (*n* = 151)	Combination (*n* = 157)	*p* *
Sex				
Male	101 (32.8%)	40 (26.5%)	61 (38.9%)	0.0209
Female	207 (67.2%)	111 (73.5%)	96 (61.1%)	
Fitzpatrick skin type				
III	245 (79.6%)	122 (80.8%)	123 (78.3%)	0.5940
IV	63 (20.5%)	29 (19.2%)	34 (21.7%)	
Type				
Retentional	54 (17.5%)	40 (26.5%)	14 (8.9%)	0.0001
Inflammatory	71 (23.1%)	36 (23.8%)	35 (22.3%)	
Mixed	183 (59.4%)	75 (49.7%)	108 (68.8%)	
Sensitive				
No	164 (53.2%)	74 (49.0%)	90 (57.3%)	0.1436
Yes	144 (46.8%)	77 (51.0%)	67 (42.7%)	
Acne scar				
No	219 (71.1%)	114 (75.5%)	105 (66.9%)	0.0953
Yes	89 (28.9%)	37 (24.5%)	52 (33.1%)	
Post‐inflammatory erythema				
No	13 (4.2%)	10 (6.6%)	3 (1.9%)	0.0398
Yes	295 (95.8%)	141 (93.4%)	154 (98.1%)	
Post‐inflammatory hyperpigmentation				
No	103 (33.4%)	53 (35.1%)	50 (31.8%)	0.5453
Yes	205 (66.6%)	98 (64.9%)	107 (68.2%)	

(B)	Sensitive				Non‐sensitive			
	Total (*n* = 144)	Monotherapy (*n* = 77)	Combination (*n* = 67)	*p* *	Total (*n* = 164)	Monotherapy (*n* = 74)	Combination (*n* = 90)	*p* *
Sex								
Male	33 (22.9%)	16 (20.8%)	17 (25.4%)	0.5130	68 (41.5%)	24 (32.4%)	44 (48.9%)	0.0333
Female	111 (77.1%)	61 (79.2%)	50 (74.6%)		96 (58.5%)	50 (67.6%)	46 (51.1%)	
Fitzpatrick skin type								
III	118 (81.9%)	64 (83.1%)	54 (80.6%)	0.6950	127 (77.4%)	58 (78.4%)	69 (76.7%)	0.7941
IV	26 (18.1%)	13 (16.9%)	13 (19.4%)		37 (22.6%)	16 (21.6%)	21 (23.3%)	
Type								
Retentional	29 (20.1%)	18 (23.4%)	11 (16.4%)	0.5700	25 (15.3%)	22 (29.7%)	3 (3.3%)	< 0.0001
Inflammatory	36 (25.0%)	19 (24.7%)	17 (25.4%)		35 (21.3%)	17 (23.0%)	18 (20.0%)	
Mixed	79 (54.9%)	40 (51.9%)	39 (58.2%)		104 (63.4%)	35 (47.3%)	69 (76.7%)	
Acne scar								
No	115 (79.9%)	58 (75.3%)	57 (85.1%)	0.1456	104 (63.4%)	56 (75.7%)	48 (53.3%)	0.0031
Yes	29 (20.1%)	19 (24.7%)	10 (14.9%)		60 (36.6%)	18 (24.3%)	42 (46.7%)	
Post‐inflammatory erythema								
No	4 (2.8%)	3 (3.9%)	1 (1.5%)	0.3813	9 (5.5%)	7 (9.5%)	2 (2.2%)	0.0429
Yes	140 (97.2%)	74 (96.1%)	66 (98.5%)		155 (94.5%)	67 (90.5%)	88 (97.8%)	
Post‐inflammatory hyperpigmentation								
No	42 (29.2%)	19 (24.7%)	23 (34.3%)	0.2037	61 (37.2%)	34 (45.9%)	27 (30%)	0.0355
Yes	102 (70.8%)	58 (75.3%)	44 (65.7%)		103 (62.8%)	40 (54.1%)	63 (70%)	
Main causes of acne								
Acne cosmetica	85 (59%)	41 (53.2%)	44 (65.7%)	0.5905	1 (100%)	0 (0%)	1 (100%)	.
Post‐Procedures (Feeling, Botox, Skin Booster)	59 (41%)	36 (46.8%)	23 (34.3%)		0 (0%)	0 (0%)	0 (0%)	
Adverse skin reaction								
No	64 (44.4%)	16 (20.8%)	48 (71.6%)	< 0.0001	164 (100%)	74 (100%)	90 (100%)	.
Yes	80 (55.6%)	61 (79.2%)	19 (28.4%)		0 (0%)	0 (0%)	0 (0%)	

* Chi‐squared test.

The sensitive patient subgroup comprised 144 patients (46.8%), and the non‐sensitive patient subgroup comprised 164 patients (53.2%). Within the sensitive patient subgroup, there were 77 patients on DC monotherapy and 67 patients on combination therapy (29 in the drug combination group, 38 in the physical modality combination group). Within the non‐sensitive patient subgroup, there were 74 patients on DC monotherapy and 90 patients on combination therapy (51 in the drug combination group, 39 in the physical modality combination group) (Table 1B).

Significant differences in patient characteristics were observed between the DC monotherapy and combination therapy groups at baseline. The DC monotherapy group had a significantly higher mean age (31.28 ± 8.97 years vs. 27.77 ± 8.78 years, *p* = 0.0004) compared to the combination therapy group. Conversely, the combination therapy group had significantly higher baseline overall acne severity (GEA: 2.36 ± 0.49 vs. 2.01 ± 0.45, *p* < 0.0001), total acne lesion count (33.16 ± 15.06 units vs. 22.85 ± 10.26 units, *p* < 0.0001), and sebum secretion level (5.81 ± 1.76 points vs. 5.28 ± 1.58 points, *p* = < 0.0001) than the DC monotherapy group (Table 2).

**TABLE 2:** Comparison of sensitivity‐related clinical parameters from baseline to Day 84 between monotherapy and combination therapy.

	Monotherapy (*n* = 151)			Combination (*n* = 157)			*p* *
	Day 0	Day 84	Difference	Day 0	Day 84	Difference	
Age (years)	31.28 ± 8.97			27.77 ± 8.78			0.0004
Erythema	1.28 ± 0.57	0.11 ± 0.31	−1.18 ± 0.6	1.34 ± 0.62	1.08 ± 0.62	−0.26 ± 0.68	0.4000
Desquamation	0.44 ± 0.55	0 ± 0	−0.44 ± 0.55	0.41 ± 0.57	0.17 ± 0.44	−0.24 ± 0.46	0.5313
Dryness	0.57 ± 0.59	0.02 ± 0.14	−0.55 ± 0.60	0.22 ± 0.43	1.06 ± 0.80	0.84 ± 0.96	< 0.0001
Itching	1.26 ± 0.64	0.04 ± 0.20	−1.23 ± 0.68	0.98 ± 0.76	1.23 ± 0.73	0.25 ± 0.91	0.0005
Tingling sensation	0.79 ± 0.67	0.01 ± 0.11	−0.78 ± 0.66	0.68 ± 0.63	0.68 ± 0.63	−0.01 ± 0.66	0.1477
Burning sensation	0.77 ± 0.68	0.01 ± 0.11	−0.76 ± 0.68	0.65 ± 0.60	0.70 ± 0.58	0.05 ± 0.67	0.1300
Pain	0.77 ± 0.69	0 ± 0	−0.77 ± 0.69	0.56 ± 0.61	0.38 ± 0.59	−0.18 ± 0.46	0.0082
Quality of life	6.55 ± 2.16	0.52 ± 0.92	−6.03 ± 2.52	6.66 ± 1.82	5.07 ± 2.66	−1.59 ± 2.21	0.6529
GEA	2.01 ± 0.45	1.05 ± 0.44	−0.96 ± 0.50	2.36 ± 0.49	1.45 ± 0.51	−0.90 ± 0.59	< 0.0001
Sebum secretion level	5.28 ± 1.58	3.43 ± 1.32	−1.85 ± 1.00	5.81 ± 1.76	3.04 ± 1.42	−2.76 ± 1.28	< 0.0001
Total acne lesion count	22.85 ± 10.26	6.27 ± 4.33	−16.58 ± 7.80	33.16 ± 15.06	14.00 ± 7.69	−19.16 ± 11.00	< 0.0001
GEA improvement		4.66 ± 0.56			4.03 ± 0.78		< 0.0001
Tolerance evaluation		3.92 ± 0.31			2.77 ± 0.96		< 0.0001

Abbreviation: GEA, Global Evaluation of Acne.

* Wilcoxon rank sum test of difference.

### 3.2 Incidence of “Acne Cosmetica” and Procedure‐Induced Acne and Its Association With Sensitive Patients

Among the 144 patients identified with initial skin sensitivity, 59.0% (*n* = 85) reported acne development attributed to cosmetic use (specifically categorized as acne cosmetica), while 41.0% (*n* = 59) experienced acne elicited by aesthetic procedures, including peeling, botulinum toxin injections, or skin booster treatments.

### 3.3 Overall Clinical Benefits and Efficacy Comparison

**FIGURE 1:**
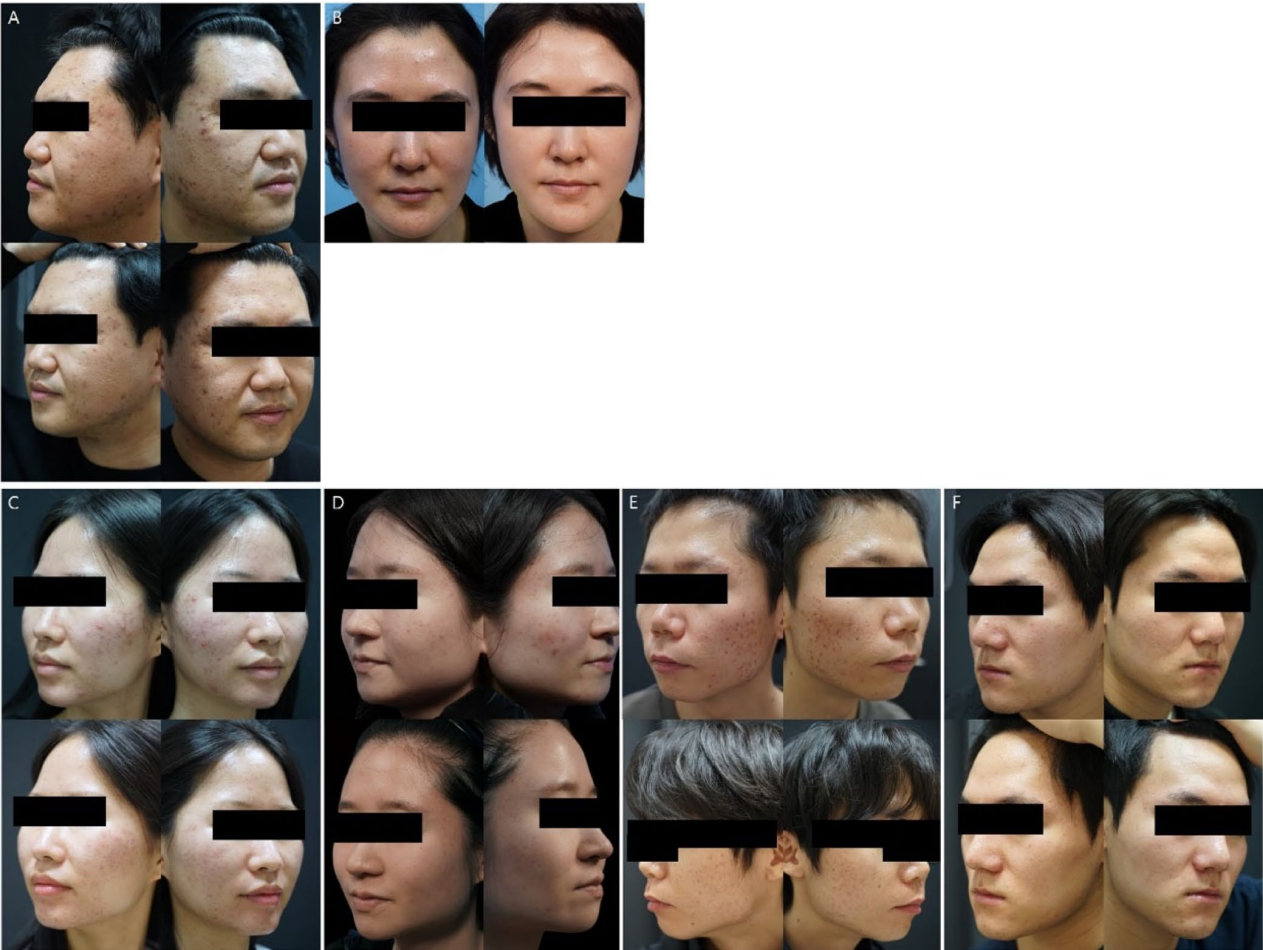
Representative clinical photographs of patients. (A) DC monotherapy (upper, before treatment; lower, after treatment). (B) Combination therapy (left, before treatment; right, after treatment). (C) Sensitive patient treated with DC monotherapy (upper, before treatment; lower, after treatment). (D) Sensitive patient treated with combination therapy (upper, before treatment; lower, after treatment). (E) Non‐sensitive patient treated with DC monotherapy (upper, before treatment; lower, after treatment). (F) Non‐sensitive patient treated with combination therapy (upper, before treatment; lower, after treatment).

**FIGURE 2:**
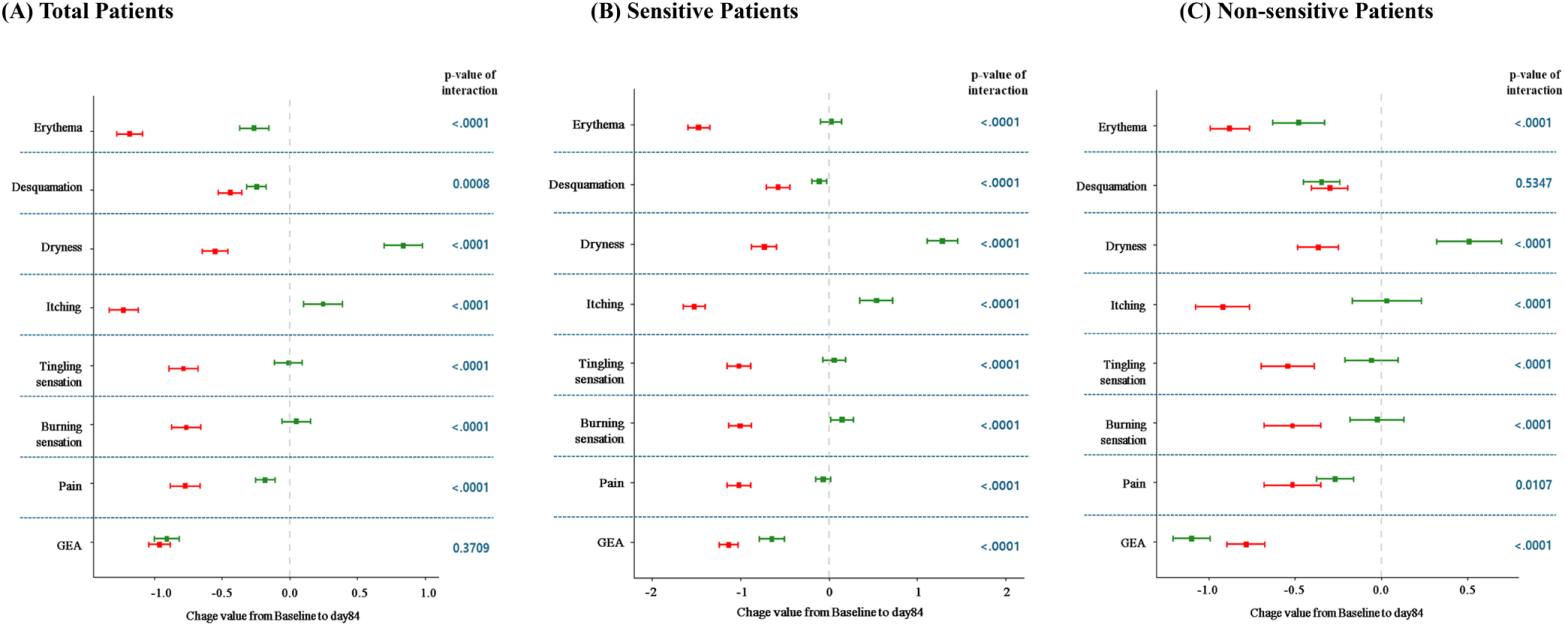
Changes in Local Skin Symptoms and Global Evaluation Scores from Baseline to Day 84 Between Treatment Groups in non‐sensitive patients. Mixed model for changes from Day 0 to Day 84; red point is monotherapy and green point is combination; line is 95% confidence interval; The *p*‐value of interaction corresponds to confirming the interaction between monotherapy and combination. GEA, Global Evaluation of Acne.

- Both DC monotherapy and combination therapy, when applied for 3 months in the overall cohort, demonstrated clinical benefits in reducing acne lesions and improving overall acne severity (Figure 1A,B).
- Acne severity: Overall acne severity improvement was significantly higher in the DC monotherapy regimen (4.66 ± 0.56) than in the combination therapy regimen (4.03 ± 0.78) (*β* = 0.64; 95% CI (0.49, 0.80); *p* < 0.0001). The reduction in total acne lesion count was also significantly greater in the combination therapy regimen than in the DC monotherapy regimen (*p* = 0.0187) (Tables 2 and 3).
- Skin sensitivity symptom: The DC monotherapy regimen showed significantly superior results compared to the combination therapy in most sensitivity index changes, such as erythema (*p* < 0.0001), desquamation (*p* = 0.0008), dryness (*p* < 0.0001), itching (*p* < 0.0001), tingling sensation (*p* < 0.0001), burning sensation (*p* < 0.0001) and pain (*p* < 0.0001) (Figure 2A).
- Sebum secretion level: The reduction in sebum secretion was significantly greater in the combination therapy regimen than in the DC monotherapy regimen (*p* < 0.0001) (Table 3).
- Tolerability: The DC monotherapy regimen demonstrated significantly higher tolerability scores than the combination regimen (*p* < 0.0001) (Table 3).
- Quality of life (CADI): The total CADI score was significantly lower and showed greater improvement in the DC monotherapy compared to the combination therapy (*p* < 0.0001) (Table 3).

**TABLE 3:** Comparison of clinical and dermatological outcomes between monotherapy and combination therapy groups.

Dependent variable	Group	*β* (95% CI)	*p* *	Interaction *p* **
Quality of life	Monotherapy	−6.03 (−6.41, −5.66)	< 0.0001	< 0.0001
	Combination	−1.59 (−1.94, −1.24)	< 0.0001	
Sebum secretion level	Monotherapy	−1.85 (−2.02, −1.69)	< 0.0001	< 0.0001
	Combination	−2.76 (−2.98, −2.55)	< 0.0001	
Total acne lesion count	Monotherapy	−16.58 (−17.84, −15.33)	< 0.0001	0.0187
	Combination	−19.16 (−20.89, −17.43)	< 0.0001	
GEA improvement***	Monotherapy (ref. Combination)	0.64 (0.49, 0.80)	< 0.0001	
Tolerance evaluation***	Monotherapy (ref. Combination)	1.21 (1.05, 1.37)	< 0.0001	

Abbreviation: GEA, Global Evaluation of Acne.

* Mixed model for changes from Day 0 to Day 84.

** Mixed mode for interaction analysis.

*** Multiple linear model adjusted age, sex.

### 3.4 Treatment Efficacy Based on Initial Skin Sensitivity

#### 3.4.1 Treatment Outcomes in the Sensitive Patient Subgroup

- Among sensitive skin patients (*n* = 144), 77 received the DC monotherapy regimen and 67 received the combination therapy regimen. Within the combination therapy group, physical modality combination therapy (*n* = 38, 56.7%) was used more frequently than drug combination therapy (*n* = 29, 43.3%).
- Acne severity: Among patients with sensitive skin, improvement in overall acne severity was observed in both the DC monotherapy and combination therapy groups (Figure 1C,D). However, the degree of improvement was significantly higher in the DC monotherapy (5.00 ± 0.00) than in the combination therapy (3.33 ± 0.47) (*β* = 1.66; 95% CI (1.55, 1.76); *p* < 0.0001). The reduction in total acne lesion count was significantly greater in the DC monotherapy than in the combination therapy (*p* = 0.0088) (Tables 4 and 5).
- Sebum secretion level: The reduction in sebum secretion was slightly smaller in the DC monotherapy group than in the combination therapy group; however, this difference was not statistically significant (*p* = 0.1145) (Table 5).
- Skin sensitivity symptom: The DC monotherapy regimen showed significantly superior results compared to the combination therapy in most sensitivity index changes, such as erythema (*p* < 0.0001), desquamation (*p* < 0.0001), dryness (*p* < 0.0001), itching (*p* < 0.0001), tingling sensation (*p* < 0.0001), burning sensation (*p* < 0.0001), and pain (*p* < 0.0001) (Figure 2B).
- Tolerability: Tolerability evaluation indicated that the DC monotherapy regimen had significantly higher tolerability scores than the combination therapy regimen (*p* < 0.0001) (Table 5).
- Quality of life (CADI): The total CADI score was significantly lower and showed greater improvement in the DC monotherapy compared to the combination therapy (*p* < 0.0001) (Table 5).

**TABLE 4:** Comparison of sensitivity‐related clinical parameters from baseline to Day 84 between monotherapy and combination therapy in sensitive patients.

	Monotherapy (*n* = 77)			Combination (*n* = 67)			*p* *
	Day 0	Day 84	Difference	Day 0	Day 84	Difference	
Age (years)	31.83 ± 7.74			29.01 ± 8.66			0.0348
Erythema	1.52 ± 0.53	0.05 ± 0.22	−1.47 ± 0.55	1.33 ± 0.53	1.36 ± 0.51	0.03 ± 0.49	0.0351
Desquamation	0.57 ± 0.59	0 ± 0	−0.57 ± 0.59	0.40 ± 0.60	0.30 ± 0.55	−0.10 ± 0.35	0.0566
Dryness	0.73 ± 0.62	0 ± 0	−0.73 ± 0.62	0.16 ± 0.37	1.45 ± 0.66	1.28 ± 0.69	< 0.0001
Itching	1.52 ± 0.55	0 ± 0	−1.52 ± 0.55	1.12 ± 0.73	1.66 ± 0.48	0.54 ± 0.77	0.0010
Tingling sensation	1.01 ± 0.57	0 ± 0	−1.01 ± 0.57	0.93 ± 0.66	0.99 ± 0.59	0.06 ± 0.52	0.4222
Burning sensation	1.00 ± 0.56	0 ± 0	−1.00 ± 0.56	0.82 ± 0.60	0.97 ± 0.52	0.15 ± 0.53	0.0715
Pain	1.01 ± 0.57	0 ± 0	−1.01 ± 0.57	0.75 ± 0.66	0.69 ± 0.68	−0.06 ± 0.34	0.0098
Quality of life	7.62 ± 1.87	0.10 ± 0.53	−7.52 ± 1.90	7.03 ± 1.79	6.99 ± 1.88	−0.04 ± 0.51	0.0358
GEA	2.06 ± 0.44	0.94 ± 0.34	−1.13 ± 0.47	2.30 ± 0.46	1.66 ± 0.48	−0.64 ± 0.57	0.0036
Sebum secretion level	5.18 ± 1.59	3.21 ± 1.39	−1.97 ± 1.00	5.37 ± 1.90	3.10 ± 1.50	−2.27 ± 1.23	0.2108
Total acne lesion count	24.29 ± 9.49	5.10 ± 2.78	−19.18 ± 7.91	33.04 ± 13.01	17.52 ± 7.23	−15.52 ± 8.61	< 0.0001
GEA improvement		5.00 ± 0.00			3.33 ± 0.47		< 0.0001
Tolerance evaluation		4.00 ± 0.00			1.96 ± 0.64		< 0.0001

Abbreviation: GEA, Global Evaluation of Acne.

* Wilcoxon rank sum test.

**TABLE 5:** Comparison of clinical and dermatological outcomes between monotherapy and combination therapy groups in sensitive patients.

Dependent variable	Group	*β* (95% CI)	*p* *	Interaction *p* **
Quality of life	Monotherapy	−7.52 (−7.95, −7.09)	< 0.0001	< 0.0001
	Combination	−0.04 (−0.17, 0.08)	0.4710	
Sebum secretion level	Monotherapy	−1.97 (−2.20, −1.75)	< 0.0001	0.1145
	Combination	−2.27 (−2.57, −1.97)	< 0.0001	
Total acne lesion count	Monotherapy	−19.18 (−20.98, −17.39)	< 0.0001	0.0088
	Combination	−15.52 (−17.62, −13.42)	< 0.0001	
GEA improvement***	Monotherapy (ref. Combination)	1.66 (1.55, 1.76)	< 0.0001	
Tolerance evaluation***	Monotherapy (ref. Combination)	2.07 (1.98, 2.21)	< 0.0001	

Abbreviation: GEA, Global Evaluation of Acne.

* Mixed model for changes from Day 0 to Day 84.

** Mixed mode for interaction analysis.

*** Multiple linear model adjusted age, sex.

#### 3.4.2 Treatment Outcomes in the Non‐Sensitive Patient Subgroup

In the patient group with no skin sensitivity at the study's commencement (*n* = 164), patients were divided into the DC monotherapy regimen (*n* = 74) and the combination regimen (*n* = 90).

- Acne severity: Among patients without initial skin sensitivity, improvement in overall acne severity was observed in both the DC monotherapy and combination therapy groups (Figure 1E,F). However, the degree of improvement was significantly higher in the combination therapy regimen (4.54 ± 0.50) than in the DC monotherapy regimen (4.31 ± 0.64) (*β* = 0.25; 95% CI (0.07, 0.43); *p* = 0.0079). The reduction in total acne lesion count was also significantly greater in the combination therapy regimen than in the DC monotherapy regimen (*p* < 0.0001) (Tables 6 and 7)
- Sebum secretion level: The reduction in sebum secretion was significantly greater in the combination therapy regimen than in the DC monotherapy regimen (*p* < 0.0001) (Table 7).
- Skin sensitivity symptom: The DC monotherapy regimen showed significantly superior results compared to the combination therapy in most sensitivity index changes, such as erythema (*p* < 0.0001), dryness (*p* < 0.0001), itching (*p* < 0.0001), tingling sensation (*p* < 0.0001), burning sensation (*p* < 0.0001), and pain (*p* = 0.0107) (Figure 2C).
- Tolerability: The DC monotherapy regimen demonstrated significantly higher tolerability scores than the conventional therapy regimen (*p* < 0.0001) (Table 7).
- Quality of life (CADI): The total CADI score was significantly lower and showed greater improvement in the DC monotherapy compared to the combination therapy (*p* < 0.0001) (Table 7).

**TABLE 6:** Comparison of sensitivity‐related clinical parameters from baseline to Day 84 between monotherapy and combination therapy in non‐sensitive patients.

	Monotherapy (*n* = 74)			Combination (*n* = 90)			*p* *
	Day 0	Day 84	Difference	Day 0	Day 84	Difference	
Age (years)	30.7 ± 10.11			26.84 ± 8.80			0.0080
Erythema	1.04 ± 0.51	0.16 ± 0.37	−0.88 ± 0.50	1.34 ± 0.67	0.87 ± 0.60	−0.48 ± 0.72	0.0012
Desquamation	0.30 ± 0.46	0 ± 0	−0.30 ± 0.46	0.41 ± 0.54	0.07 ± 0.29	−0.34 ± 0.50	0.1941
Dryness	0.41 ± 0.52	0.04 ± 0.20	−0.36 ± 0.51	0.27 ± 0.47	0.78 ± 0.78	0.51 ± 1.00	0.0673
Itching	1.00 ± 0.62	0.08 ± 0.27	−0.92 ± 0.68	0.88 ± 0.76	0.91 ± 0.73	0.03 ± 0.95	0.1713
Tingling sensation	0.57 ± 0.68	0.03 ± 0.16	−0.54 ± 0.67	0.50 ± 0.55	0.44 ± 0.56	−0.06 ± 0.74	0.7754
Burning sensation	0.54 ± 0.71	0.03 ± 0.16	−0.51 ± 0.71	0.52 ± 0.57	0.50 ± 0.55	−0.02 ± 0.75	0.7627
Pain	0.51 ± 0.71	0 ± 0	−0.51 ± 0.71	0.42 ± 0.54	0.16 ± 0.39	−0.27 ± 0.51	0.6866
Quality of life	5.43 ± 1.87	0.95 ± 1.03	−4.49 ± 2.13	6.39 ± 1.81	3.64 ± 2.23	−2.74 ± 2.28	0.0009
GEA	1.95 ± 0.46	1.16 ± 0.50	−0.78 ± 0.48	2.40 ± 0.51	1.30 ± 0.48	−1.10 ± 0.52	< 0.0001
Sebum secretion level	5.39 ± 1.58	3.66 ± 1.22	−1.73 ± 1.00	6.13 ± 1.59	3.00 ± 1.37	−3.13 ± 1.38	< 0.0001
Total acne lesion count	21.36 ± 10.87	7.49 ± 5.25	−13.88 ± 6.73	33.24 ± 16.50	11.38 ± 6.97	−21.87 ± 11.82	< 0.0001
GEA improvement		4.31 ± 0.64			4.54 ± 0.50		0.0247
Tolerance evaluation		3.84 ± 0.44			3.38 ± 0.65		< 0.0001

Abbreviation: GEA, Global Evaluation of Acne.

* Wilcoxon rank sum test.

**TABLE 7:** Comparison of clinical and dermatological outcomes between monotherapy and combination therapy groups in non‐sensitive patients.

Dependent variable	Group	*β* (95% CI)	*p* *	Interaction *p* **
Quality of life	Monotherapy	−4.49 (−4.98, −3.99)	< 0.0001	< 0.0001
	Combination	−2.74 (−3.22, −2.27)	< 0.0001	
Sebum secretion level	Monotherapy	−1.73 (−1.96, −1.50)	< 0.0001	< 0.0001
	Combination	−3.13 (−3.42, −2.85)	< 0.0001	
Total acne lesion count	Monotherapy	−13.88 (−15.44, −12.32)	< 0.0001	< 0.0001
	Combination	−21.87 (−24.34, −19.39)	< 0.0001	
GEA improvement***	Combination (ref. Monotherapy)	0.25 (0.07, 0.43)	0.0079	
Tolerance evaluation***	Monotherapy (ref. Combination)	0.48 (0.30, 0.66)	< 0.0001	

Abbreviation: GEA, Global Evaluation of Acne.

* Mixed model for changes from Day 0 to Day 84.

** Mixed mode for interaction analysis.

*** Multiple linear model adjusted age, sex.

### 3.5 Efficacy of Physical Modality Combination Within the DC‐Integrated Conventional Therapy Group

- Combination therapy group of sensitive patients: The physical modality combination group showed significantly higher tolerability (*p* = 0.0001) and significantly higher GEA improvement (*p* < 0.0001) compared to the drug combination group (Table S1).
- Combination therapy group of non‐sensitive patients: The physical modality combination group showed significantly higher tolerability (*p* < 0.0001) but significantly lower GEA improvement (*p* < 0.0001) compared to the drug combination group (Table S1).

### 3.6 Irritation‐Related Adverse Events and the Efficacy of “Stepwise Progressive Introduction” Guideline

During the study period, no irritation‐related adverse events were reported in the initially non‐sensitive patient subgroup. However, in the initially sensitive patient subgroup, irritation‐related adverse events occurred in 80 patients (55.6%), demonstrating a statistically significant difference compared to the initially non‐sensitive group (OR: 410.61, 95% CI: 24.88 ~ 999.99, *p* < 0.0001). Among the 80 patients in the initially sensitive patient subgroup who experienced adverse events, 71 patients (88.75%) were classified as “mild” (e.g., irritation), and 9 patients (11.25%) were classified as “moderate,” and all irritation‐related adverse events resolved after applying stepwise progressive‐introduction measures (e.g., reducing the dose of the standard DC regimen). This improvement was clinically observed at the Day 84 visit.

## 4 Discussion

This study shows that a specific DC monotherapy regimen can offer comparable, and in some aspects superior, clinical efficacy to combination therapy regimens for acne management. Improvements in GEA, total acne lesion count, and sebum secretion levels indicate that effective acne management can be achieved without excessive drug use or complex treatment protocols. This represents an important step toward patient‐centric, minimal intervention strategies, offering a practical alternative, especially given the high rates of treatment discontinuation associated with adverse events from conventional pharmacological agents.

However, before interpreting the results, baseline differences between groups should be considered. Owing to the non‐randomized, open‐label design, the DC monotherapy group tended to have lower baseline severity (lower GEA, fewer total lesion counts, lower sebum secretion level) than the combination group. This raises the possibility of selection bias and confounding by indication, implying that the observed superiority of DC monotherapy may partly reflect baseline imbalances rather than a true treatment effect. Further confirmation in prospective randomized trials using stratified randomization by initial sensitivity and baseline severity with covariate‐adjusted analyses might be needed. These findings should therefore be interpreted as supporting a sensitivity‐guided treatment approach rather than indicating the universal superiority of a single treatment regimen.

Our results show that a patient's initial skin sensitivity is a critical clinical indicator that should be evaluated before initiating acne treatment. In our cohort, more than half of patients with initial sensitivity subsequently developed irritation‐related adverse events, indicating a persistent sensitive‐skin profile. In this subgroup, DC monotherapy was superior to combination regimens for improving acne severity, tolerability, and quality of life. These findings suggest that sensitive skin might be inherently more susceptible to drug ingredients or intensive procedures that can impair skin barrier and exacerbate inflammation. Accordingly, initiating treatment with a structured DC monotherapy may provide a more balanced approach between efficacy and tolerability in sensitive patients. Clinical observations further support a gradual, stepwise introduction strategy. In patients who experienced irritation, reducing application amount and gradually adjusting frequency, treated area, and total load improved tolerability and adherence, with further improvement in acne severity and quality of life. These findings emphasize the importance of individualized product use under dermatologic guidance, particularly in patients with sensitive skin. Taken together, these results underscore the importance of baseline skin sensitivity in guiding acne treatment selection and provide preliminary support for a sensitivity‐guided approach. We therefore advocate integrating an individualized acne management strategy such as standardized, sensitivity‐guided DC regimens into future guidance to enhance both treatment effectiveness and patient satisfaction.

The strength of our study is that we enrolled patients across primary, secondary, and tertiary care to capture a diverse real‐world acne population, and we expanded the scope from a simple DC‐monotherapy versus combination therapy comparison to a sensitivity‐stratified evaluation.

This study has several limitations. First, as it was designed as a non‐randomized, open‐label observational study without a formal control group, the potential for selection bias and residual confounding cannot be excluded. Furthermore, potential confounding factors, including baseline acne severity, prior treatments, hormonal status, lifestyle factors, and concurrent skincare practices, may have influenced the observed outcomes and could not be fully controlled. Second, the study population was limited to Korean patients with acne, and thus does not fully capture potential differences in acne characteristics or cosmetic use patterns across different ethnicities. Third, initial skin sensitivity was defined based on patients' self‐reports of perceived irritation or adverse reactions to prior cosmetic products or procedures, rather than a formally validated sensitive‐skin scale, which may introduce subjectivity and potential recall bias.

## 5 Conclusion

This study suggests that baseline skin sensitivity may represent an important determinant of treatment selection and outcomes in acne management, and that clinically validated DCs may serve as a meaningful component of personalized treatment strategies.

## Author Contributions

J.H.H., J.M.B., and E.B. conceived and designed the study. J.H.H., H.J.J., T.I.K., Y.H.Y., Y.J.W., H.K., J.Y.K., and J.M.B. recruited participants, acquired clinical data, and managed the study. S.J.J. performed the statistical analyses. J.H.H. and J.M.B. interpreted the data. J.H.H. drafted the first version of the manuscript. H.J.J., T.I.K., Y.H.Y., Y.J.W., H.K., J.Y.K., S.J.J., E.B., and J.M.B. critically revised the manuscript. All authors have read and approved the final manuscript.

## Funding

The authors have nothing to report.

## Ethics Statement

This study was conducted in accordance with the Declaration of Helsinki and was approved by the ethics committee of Seoul Saint Mary's Hospital (approval No. KC24ESSI0480; approval date: 22 Nov 2024).

## Consent

Written informed consent for the publication of all clinical photographs was obtained from the patient(s) (and, where applicable, from their legal guardians).

## Conflicts of Interest

Eunsun Baek is an employee of La Roche‐Posay Laboratoire Dermatologique. The other authors declare no conflicts of interest.

## Supporting information


**Table S1:** Comparison of Clinical and Dermatological Outcomes Between Different Conventional Modalities Used in the DC‐Integrated Therapy Group.

## Data Availability

The data that support the findings of this study are available on request from the corresponding author. The data are not publicly available due to privacy or ethical restrictions.
