# Supplementary material for: Personalized Approach for Acne Management With Dermocosmetics According to Initial Skin Sensitivity
Source: J Cosmet Dermatol. 2026 Apr 8;25(4):e70831. doi: 10.1111/jocd.70831 (PMC13059542; doi:10.1111/jocd.70831)
Supplement: Supplementary file 1 — Table S1: Comparison of Clinical and Dermatological Outcomes Between Different Conventional Modalities Used in the DC‐Integrated Therapy Group. [file JOCD-25-e70831-s001.docx]

**Table S1. Comparison of Clinical and Dermatological Outcomes Between Different Conventional Modalities Used in the DC-Integrated Therapy Group.**

|  | **Group** | **Sensitive patients** | | | **Non-sensitive patients** | | |
| --- | --- | --- | --- | --- | --- | --- | --- |
|  |  | **β (95% CI)** | ***p*-value*** | **Interaction  *p*-value**** | **β (95% CI)** | ***p*-value*** | **Interaction  *p*-value**** |
| Erythema | Drug | 0.10(-0.11, 0.32) | 0.3262 | 0.2877 | -0.08(-0.29, 0.13) | 0.4552 | <.0001 |
|  | Physical Modality | -0.03(-0.17, 0.12) | 0.7100 |  | -1.00(-1.00, -0.99) | <.0001 |  |
| Desquamation | Drug | 0.03(-0.04, 0.11) | 0.3262 | 0.0042 | -0.22(-0.35, -0.09) | 0.0016 | 0.0047 |
|  | Physical Modality | -0.21(-0.35, -0.07) | 0.0034 |  | -0.51(-0.66, -0.36) | <.0001 |  |
| Dryness | Drug | 1.41(1.20, 1.63) | <.0001 | 0.1805 | 1.24(1.07, 1.40) | <.0001 | <.0001 |
|  | Physical Modality | 0.18(0.96, 1.41) | <.0001 |  | -0.44(-0.60, -0.27) | <.0001 |  |
| Itching | Drug | 0.59(0.29, 0.88) | 0.0004 | 0.6513 | 0.65(0.45, 0.85) | <.0001 | <.0001 |
|  | Physical Modality | 0.50(0.25, 0.75) | 0.0003 |  | -0.77(-0.94, -0.60) | <.0001 |  |
| Tingling sensation | Drug | 0.24(0.08, 0.41) | 0.0060 | 0.0112 | 0.37(0.20, 0.54) | <.0001 | <.0001 |
|  | Physical Modality | -0.08(-0.26, 0.10) | 0.3729 |  | -0.62(-0.78, -0.46) | <.0001 |  |
| Burning sensation | Drug | 0.24(0.08, 0.41) | 0.0058 | 0.2160 | 0.41(0.24, 0.58) | <.0001 | <.0001 |
|  | Physical Modality | 0.08(-0.11, 0.27) | 0.4128 |  | -0.59(-0.75, -0.43) | <.0001 |  |
| Pain | Drug | 0.07(-0.03, 0.17) | 0.1613 | 0.0063 | -0.06(-0.18, 0.06) | 0.3221 | <.0001 |
|  | Physical Modality | -0.16(-0.28, -0.04) | 0.0124 |  | -0.54(-0.70, -0.38) | <.0001 |  |
| Quality of life | Drug | 0.07(-0.07, 0.21) | 0.3262 | 0.1082 | -1.53(-2.05, -1.01) | <.0001 | <.0001 |
|  | Physical Modality | -0.13(-0.06, 0.32) | 0.1688 |  | -4.33(-4.91, -3.76) | <.0001 |  |
| GEA | Drug | -0.59(-0.80, -0.37) | <.0001 | 0.4894 | -1.22(-1.38, -1.05) | <.0001 | 0.0150 |
|  | Physical Modality | -0.68(-0.87, -0.50) | <.0001 |  | -0.95(-1.08, -0.82) | <.0001 |  |
| Sebum secretion level | Drug | -2.14(-2.54, -1.73) | <.0001 | 0.4499 | -3.31(-3.65, -2.98) | <.0001 | 0.1560 |
|  | Physical Modality | -2.37(-2.81, -1.93) | <.0001 |  | -2.90(-3.41, -2.39) | <.0001 |  |
| Acne lesion number | Drug | -14.55(-17.98, -11.12) | <.0001 | 0.4243 | -25.04(-28.61, -21.47) | <.0001 | 0.0031 |
|  | Physical Modality | -16.26(-19.01, -13.52) | <.0001 |  | -17.72(-20.69, -14.75) | <.0001 |  |
| GEA improvement******* | Physical Modality  (ref. Drug) | 0.63(0.43, 0.83) | <.0001 | . | -0.50(-0.69, -0.31) | <.0001 | . |
| Tolerance evaluation******* | Physical Modality  (ref. Drug) | 0.62(0.31, 0.92) | 0.0001 | . | 1.07(0.91, 1.23) | <.0001 | . |

*Mixed model for changes from day 0 to day 84; **Mixed mode for interaction analysis; ***Multiple linear model adjusted age, sex

GEA, Global Evaluation of Acne
